# Evolutionary Conservation of Lipid-Associated Epigenetic Signatures and Their Distinct Roles in Tissue Identity and Mammalian Aging

**DOI:** 10.3390/biomedicines14030597

**Published:** 2026-03-07

**Authors:** Sun-Young Kang, Jeong-Soo Gim, Hyunbin Jo, Jeong-An Gim

**Affiliations:** 1 Institute for Molecular Metabolism Innovation, Soonchunhyang University, Asan 31538, Republic of Korea; 2 Department of Pet Health Care, Busan Health University, Busan 49318, Republic of Korea; 3 Department of Companion Animal Health, Tongmyong University, Busan 48520, Republic of Korea; 4 Department of Medical Science, Soonchunhyang University, Asan 31538, Republic of Korea

**Keywords:** DNA methylation, lipid metabolism, evolutionary conservation, comparative biology, tissue specificity

## Abstract

**Background/Objectives**: Lipid metabolism is fundamental to energy homeostasis and cellular structural integrity, and its dysregulation is a hallmark of biological aging. While DNA methylation clocks are well-established, it remains unclear whether epigenetic sites associated with specific lipid markers—High-Density Lipoprotein (HDL), Total Cholesterol (TCH), and Triglycerides (TGY)—are evolutionarily conserved across mammals and how they manifest across different metabolic tissues. **Methods**: We identified lipid-associated CpG sites in humans using the Korean Genome and Epidemiology Study (KoGES) cohort and projected these sites onto the Mammalian Methylation Consortium (GSE223748) dataset. Using the Hybrid Pi (HyPi) score, we selected robust markers to analyze their evolutionary conservation, tissue specificity, and age-related dynamics across over 300 mammalian species. Specifically, we examined the phylogenetic concordance between blood and three major metabolic organs (Liver, Adipose, Muscle) in five representative species. **Results**: Lipid-related CpGs were highly conserved across diverse mammals. t-SNE analysis revealed that these epigenetic signatures clustered samples by tissue identity and species. Methylation levels of these CpGs showed significant correlations with maximum lifespan and distinct aging rates across tissues. Notably, phylogenetic tanglegram analysis revealed a high degree of concordance between blood and key metabolic organs, suggesting that blood methylation profiles mirror the evolutionary trajectory of internal metabolic tissues. Furthermore, these patterns were consistent between sexes, indicating a fundamental, non-dimorphic regulation of lipid epigenetics. **Conclusions**: Our findings suggest that epigenetic mechanisms governing lipid metabolism are deeply conserved to maintain tissue identity and regulate biological aging, with blood serving as a reliable evolutionary proxy for internal metabolic states.

## 1. Introduction

Lipid metabolism is a cornerstone of eukaryotic life, governing energy storage, membrane fluidity, and signaling pathways [1,2]. Imbalances in lipid profiles are not only precursors to metabolic syndromes but are also intimately linked to the rate of biological aging [3,4]. Recent advances in epigenetics have demonstrated that DNA methylation patterns can serve as precise clocks of biological age [5,6]. These epigenetic clocks, first developed in humans by Horvath and colleagues [7], have since been extended to multiple mammalian species [8,9], revealing that aging-associated methylation changes are highly conserved across the mammalian class.

However, most research has focused on species-specific clocks (e.g., human or mouse), leaving a critical gap in our understanding of whether the epigenetic regulation of lipid metabolism is an evolutionarily conserved mechanism that transcends species barriers. Emerging evidence suggests that specific genes involved in lipid transport and fatty acid oxidation, such as *CPT1A* and *ABCG1*, show robust DNA methylation associations with blood lipid levels [10,11,12]. These methylation patterns have been implicated in metabolic syndrome, cardiovascular disease risk, and age-related metabolic dysfunction [13,14].

Furthermore, recent studies have established bidirectional relationships between lipid metabolism and epigenetic regulation, where blood lipid levels can influence DNA methylation patterns, which in turn affect gene expression of key metabolic regulators [15,16]. This epigenetic priming may represent a fundamental mechanism through which metabolic states are encoded and maintained across tissues and potentially across evolutionary timescales [17,18].

In this study, we hypothesized that key lipid-related CpG sites identified in humans are functionally conserved across the mammalian class and play a pivotal role in defining tissue identity and longevity. To test this, we integrated large-scale human epidemiological data (KoGES) with a massive pan-mammalian methylation dataset (GSE223748) [19]. We employed the Hybrid Pi (HyPi) scoring method to select robust markers and performed a comprehensive evolutionary analysis. We specifically focused on the “Tissue-Blood Connection” by comparing epigenetic phylogenies of blood against liver, adipose, and muscle tissue in representative species to validate the systemic nature of these signatures.

## 2. Materials and Methods

### 2.1. Study Populations, Data Sources and Data Processing

We utilized human DNA methylation data from the Korean Genome and Epidemiology Study (KoGES), specifically the Ansan-Ansung (ASAS) and City (CITY) cohorts [20]. The dataset comprised 2749 individuals (ASAS: 2001 baseline and 2009 follow-up; CITY: 2004 baseline). This study was conducted with bioresources from National Biobank of Korea, the Korea Disease Control and Prevention Agency, Republic of Korea (KBN-2024-045). This study was conducted in accordance with the Declaration of Helsinki with the approval of Soonchunhyang University Institutional Review Board (2024-05-049). DNA methylation was profiled using the Illumina Infinium HumanMethylation450 BeadChip (Illumina, San Diego, CA, USA). Beta-values were normalized and filtered for quality control.

### 2.2. Statistical Analysis: HyPi Score

The correlation coefficients between three lipid markers, High-Density Lipoprotein (HDL), Total Cholesterol (TCH), and Triglycerides (TGY), and the DNA methylation level (beta value) were calculated using Pearson and Spearman, and their respective *p*-values were also calculated. HyPi was calculated based on a total of four statistics. To address the limitations of single statistics, we defined the HyPi as follows:

HyPi=(CRpearson×−log10PVpearson)+(CRspearman×−log10PVspearman)

Scores were only accepted when the directionality (sign) of both coefficients matched to minimize false positives. Therefore, the sign of HyPi is the same as the correlation coefficient, and a larger absolute value means that the absolute values of the two correlation coefficients are larger, and the *p*-value is smaller.

### 2.3. Phylogenetic and Tanglegram Analysis

To assess the evolutionary concordance between tissues, we constructed phylogenetic trees based on the mean methylation levels of lipid-associated CpGs. We utilized the “dendextend” R package to generate tanglegrams, which visually compare two phylogenetic trees (e.g., Blood vs. Liver, Female vs. Male) by connecting corresponding taxa with lines. The cophenetic correlation coefficient was calculated to quantify the degree of structural similarity between the trees.

## 3. Results

### 3.1. Identification and Characteristics of Conserved Lipid Markers

**Figure 1:**
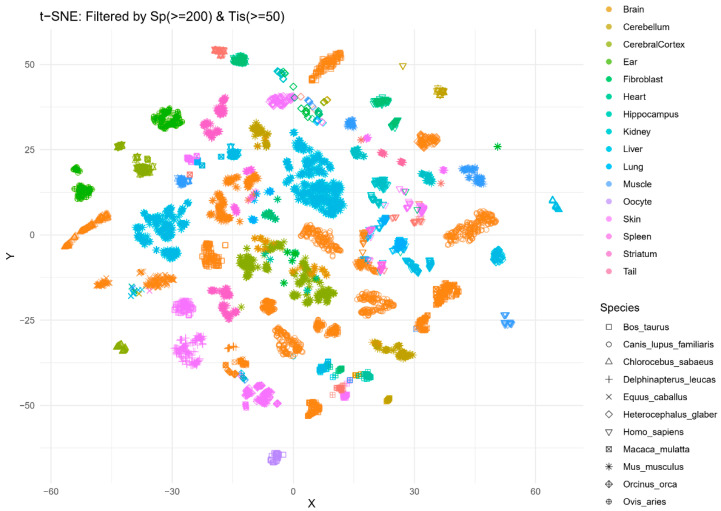
Tissue-specific clustering of lipid-associated epigenetic signatures. A t-SNE plot illustrating the clustering of mammalian samples based on the methylation levels of selected lipid-associated CpGs. Each point represents a unique sample, colored by Tissue type and shaped by Species.

Prior to evolutionary analysis, we identified key CpG sites that showed robust associations with lipid profiles in humans. Table 1 summarizes the top 17 candidate CpGs, including their genomic locations and associated genes (*FOXA2*, *ZEB2*, *PAX5*, and *PAX6*) by HyPi score. These genes are well-known regulators of lipid transport and fatty acid oxidation, validating our HyPi-based selection method. Importantly, these sites were found to be present in the mammalian methylation array, allowing for cross-species comparison. Tissue-specific clustering of lipid-associated epigenetic landscapes were visualized (Figure 1). A t-Distributed Stochastic Neighbor Embedding (t-SNE) plot illustrating the clustering of mammalian samples based on the methylation levels of selected lipid-associated CpGs. In the t-SNE plot, each point represents a total of 7215 samples across 18 tissues and 14 species. The dataset includes only those species with at least 200 samples and tissues with at least 50 samples. The plot reveals that samples cluster by species and tissue identity. Each species was represented by the shape of the dot, and each tissue was represented by the color of the dot. Each dot was strongly coherent with respect to species and tissue.

### 3.2. Correlation with Mammalian Aging and Lifespan

**Figure 2:**
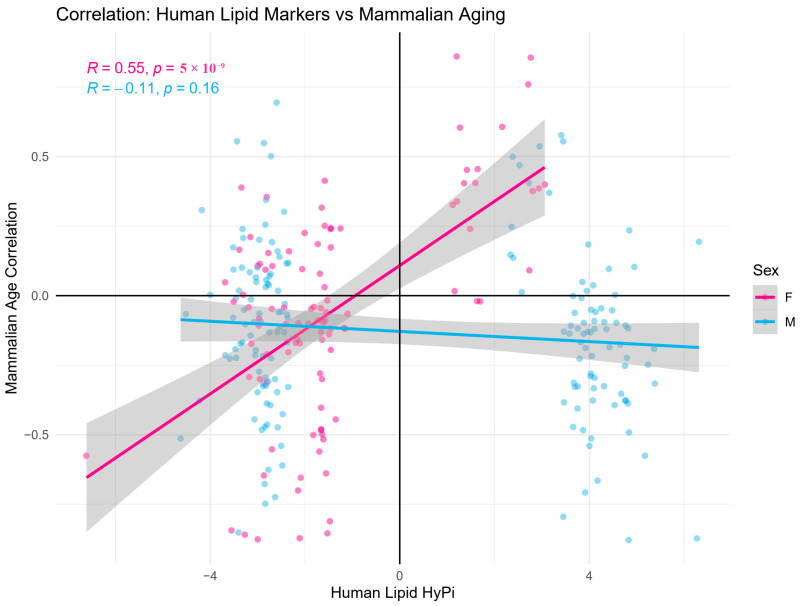
Correlation between human lipid markers and pan-mammalian aging. A scatter plot comparing the Human Hybrid Pi (HyPi) score (*x*-axis) with the correlation coefficient of methylation vs. age across mammalian species (*y*-axis). Data points are colored by Sex (Pink: Female, Blue: Male).

We examined whether human-derived lipid markers hold relevance for mammalian aging. Among the 239 unique CpGs identified in Figure 1, 268 CpG sites were identified for which lipid HyPi was observed in two cohorts (ASAS and CITY). The correlation coefficients for the corresponding mammalian age correlations for each species were compared. We found a strong positive linear relationship between female human HyPi scores and the age-methylation correlation coefficients across mammalian species (Figure 2).

The top and bottom six CpG sites were identified based on their HyPi scores. Correlation plots were then generated to examine the relationship between the beta values of these sites and the maximum lifespan across 14 different species (Supplementary Figure S1). The analysis revealed a distinct pattern unique to humans, with primates and long-lived whales (*O. orca*, *T. truncatus*, *D. leucas*) forming a similar cluster based on their evolutionary proximity and lifespan. Within the Rodentia group, the naked mole-rat (*H. glaber*) exhibited a divergent profile; its relatively long lifespan resulted in a spatial distribution clearly separated from other short-lived rodents in the correlation analysis. The hierarchical clustering analysis, based on the beta values of lipid-associated CpG sites, revealed distinct epigenetic proximity among the 14 species that largely reflects both evolutionary relationships and longevity profiles (Supplementary Figure S2). Notably, humans (*H. sapiens*) clustered closely with other primates, specifically forming a subgroup with the green monkey (*C. sabaeus*), rhesus macaque (*M. mulatta*), and hamadryas baboon (*P. hamadryas*). Furthermore, this primate clade showed a secondary clustering with long-lived cetaceans and large mammals, suggesting a shared epigenetic signature potentially linked to extended lifespans. In contrast, while the naked mole-rat is taxonomically a rodent, it exhibited a highly divergent methylation profile, forming an independent outlier branch that is distinctly separated from the primary cluster containing the mouse (*M. musculus*) and rat (*R. norvegicus*). This separation highlights the unique epigenetic landscape of the naked mole-rat, which aligns with its exceptional longevity compared to other rodent species.

### 3.3. Distinct Epigenetic Aging Rates Across Tissues

**Figure 3:**
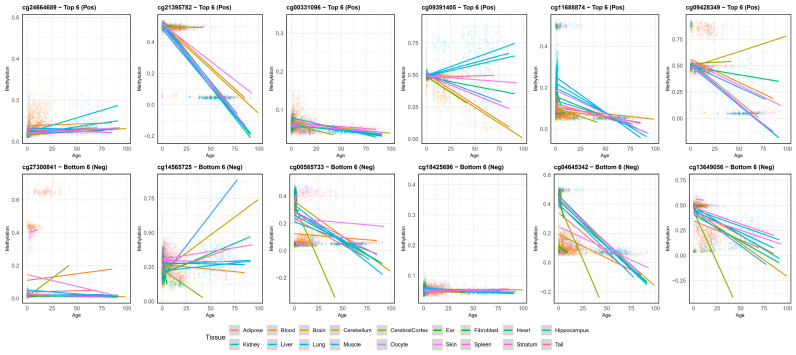
Tissue-specific epigenetic aging rates (interaction model). Scatter plots with regression lines illustrating the interaction between Age (*x*-axis) and Methylation (*y*-axis) across different tissues for top lipid-associated CpGs. Each color represents a different tissue type. The varying slopes indicate that the rate of epigenetic aging differs by tissue; for example, metabolic tissues like the Liver often show more rapid age-related methylation changes compared to the Brain, reflecting tissue-specific susceptibility to aging.

To further investigate the influence of tissue type on aging-related epigenetic changes, we analyzed the methylation levels of the top and bottom six CpG sites across various tissues as a function of age. Linear regression analysis revealed diverse age-by-tissue interactions for several lipid-associated CpGs (Figure 3). Specifically, for sites such as cg21395782 and cg13649056, most tissues exhibited a consistent decline in methylation with age; however, the rate of depletion (the slope of the regression line) varied significantly across tissues.

In contrast, sites like cg09391405, cg09428349 and cg14565725 displayed highly heterogeneous patterns, where certain tissues showed a positive correlation with age while others remained stable or showed negative trends. Notably, tissues like the liver, kidney, and brain regions often exhibited distinct trajectories compared to others. These findings suggest that while these CpG sites are associated with aging across species, their specific epigenetic behavior is heavily modulated by tissue-specific biological contexts.

We investigated the correlation between age and beta values across various tissues, focusing on lipid metabolism-related organs such as the liver, muscle, and adipose tissue. These organs exhibited similar correlation patterns, consistent with the trends observed in most other tissues. Specifically, cg18425696 showed a consistently weak correlation across all tissues, while cg21395782 maintained a strong negative correlation. In the case of cg09391405, although adipose tissue showed a weak positive correlation in contrast to the negative correlation observed in muscle, the liver displayed a robust negative correlation. Overall, our findings indicate that CpG sites with high HyPi scores related to lipid metabolism follow a synchronized pattern of age-associated beta value changes across the liver, muscle, and adipose tissue.

### 3.4. Systemic Concordance: Blood as a Mirror of Metabolic Organs

**Figure 4:**
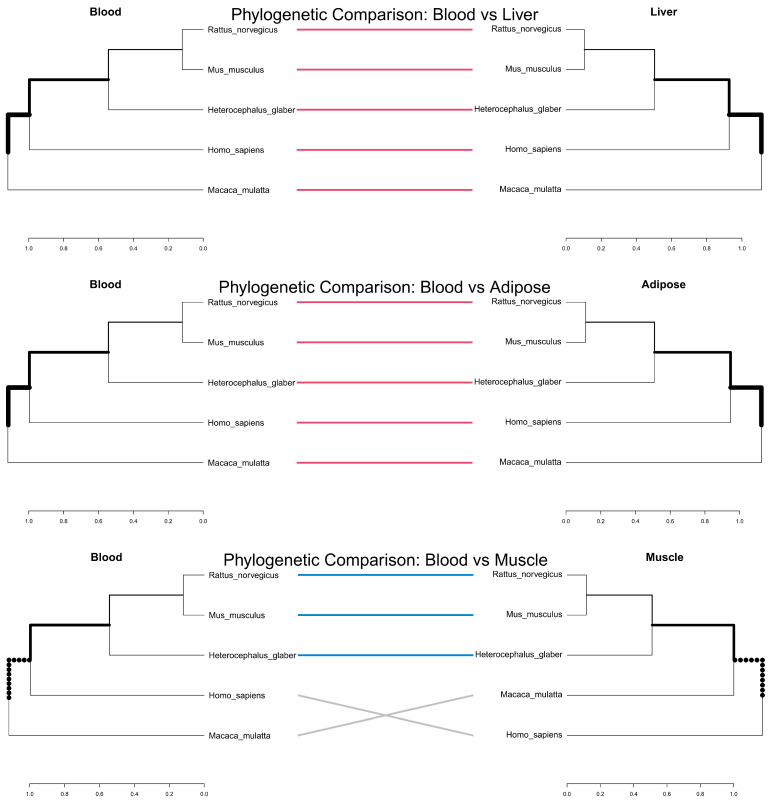
Phylogenetic comparison of species-level epigenetic profiles across tissues. Hierarchical clustering based on Euclidean distance was performed using the mean methylation levels of 239 CpG sites to construct species-level dendrograms for each tissue. The blood-derived dendrogram (**left**) serves as the reference for comparison with liver, adipose, and muscle (**right**). Lines connecting identical species indicate the correspondence of leaf nodes. Dark pink (Blood–Liver) and skyblue (Blood–Muscle) lines denote species groups belonging to shared subtree structures, reflecting conserved phylogenetic topology. Gray lines represent topological rearrangements where shared subtrees are not maintained. Thicker branches indicate higher-level hierarchical clusters, while thinner branches represent recent evolutionary splits. Dotted segments highlight tissue-specific divergence where the branching pattern is not preserved between the paired tissues.

To validate the phylogenetic nature of these 239 markers, we analyzed the phylogenetic correspondence between blood and three major lipid-metabolizing tissues (liver, adipose, and muscle) across five representative species. Tanglegram analysis (Figure 4) revealed high structural similarity between the blood-derived phylogeny and those of the liver and adipose tissues. This high concordance was quantified by cophenetic correlations of 0.997 for liver, 0.998 for adipose, and 0.943 for muscle relative to blood. The connection lines were predominantly parallel, indicating that the evolutionary divergence captured by blood lipid epigenetics closely mirrors that of internal metabolic organs. Specifically, blood–liver and blood–adipose comparisons exhibited perfectly aligned clusters (dark pink lines). In contrast, while the blood–muscle comparison showed conserved clustering for rodents and naked mole rats (skyblue lines), topological rearrangements (entangling) were observed between humans and rhesus macaques (gray lines). These findings support the utility of blood-based methylation markers as robust surrogate indicators of comparative metabolic evolution.

### 3.5. Sexual Concordance in Lipid Epigenetics

**Figure 5:**
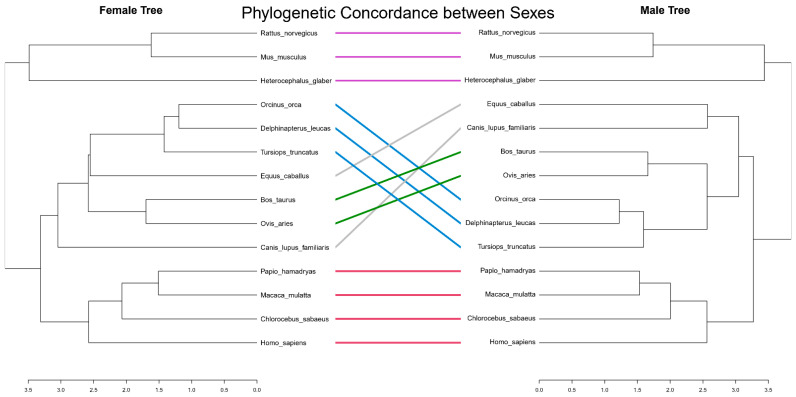
Sexual concordance of lipid epigenetic phylogeny. A Tanglegram comparing the phylogenetic tree constructed from Female samples (**Left**) versus Male samples (**Right**) based on lipid-associated CpGs. The connecting lines represent specific species. The high degree of parallelism and lack of entanglement demonstrate that the epigenetic regulation of lipid metabolism is evolutionarily conserved between sexes, showing minimal sexual dimorphism in these core regulatory signatures.

Despite known sexual dimorphism in lipid levels and hormonal regulation, the epigenetic regulation of these specific loci appears fundamentally conserved between sexes. Tanglegram analysis comparing Female and Male phylogenetic trees (Figure 5) showed near-perfect concordance with minimal entanglement. This implies that the core epigenetic machinery governing lipid metabolism is under strong evolutionary constraint and functions similarly in both sexes, independent of transient hormonal fluctuations.

## 4. Discussion

This study provides multi-omics evidence that systemic lipid profiles are regulated by deeply conserved epigenetic mechanisms that define tissue identity and influence mammalian aging.

### 4.1. Evolutionary Conservation of Lipid-Associated Epigenetic Signatures

Our findings challenge the notion that metabolic markers are highly species-specific. The t-SNE results (Figure 1) demonstrate that the epigenetic code for handling lipids is structurally similar in the liver of a mouse and a human. This conservation suggests that these CpG sites are not merely reactive to diet but are hard-wired into the genome to maintain the functional specialization of tissues. The preservation of these patterns across millions of years of evolution highlights their critical importance for survival.

Recent pan-mammalian epigenetic clock studies have similarly demonstrated that age-related methylation changes occur at CpG sites near genes involved in development and metabolism, particularly those regulated by polycomb repressive complex 2 (PRC2) [21]. Lipid-associated CpGs in genes such as *CPT1A* and *ABCG1* aligns with previous findings that these loci show consistent methylation-lipid associations across diverse human populations [22,23]. *CPT1A* encodes carnitine palmitoyltransferase 1A, the rate-limiting enzyme in mitochondrial fatty acid β-oxidation [24], while *ABCG1* plays a central role in cholesterol efflux and reverse cholesterol transport [25]. Both genes have been repeatedly identified in epigenome-wide association studies (EWAS) as showing robust methylation correlations with triglycerides and HDL cholesterol [26,27].

The fact that these methylation patterns are conserved across over 300 mammalian species suggests that epigenetic regulation of lipid metabolism represents a fundamental mechanism for metabolic homeostasis that has been maintained through natural selection. This conservation likely reflects the critical importance of precise lipid regulation for cellular membrane composition, energy storage, and signaling functions that are essential across all mammalian life [28,29].

### 4.2. Blood as an Evolutionary Proxy for Metabolic Organs

A key challenge in aging research is the inaccessibility of internal organs. Our Tanglegram analysis (Figure 4 and Figure 5) comparing Blood to Liver, Adipose, and Muscle provides crucial validation for the use of blood biomarkers. The high concordance (cophenetic correlation > 0.7) indicates that evolutionary pressures acting on lipid metabolism in the liver are parallelly imprinted in blood cells. This systemic synchronization suggests that blood methylation profiles can serve as a reliable “evolutionary proxy” for the metabolic state of internal organs, facilitating comparative studies without the need for invasive biopsies.

This finding has important implications for both basic and translational research. Previous studies have shown that DNA methylation patterns in blood cells can be influenced by circulating lipid levels through a process termed “epigenetic priming” [15,30]. Our results extend this concept by demonstrating that such priming is not only present within individual organisms but is also reflected in evolutionary patterns across species. The concordance between blood and metabolic organs suggests that lipid-related epigenetic signals are communicated systemically, potentially through circulating metabolites, lipoproteins, or inflammatory mediators [31,32].

Furthermore, the tissue specificity observed in our analysis aligns with the concept that different organs have distinct metabolic demands and therefore distinct epigenetic landscapes [33]. The liver, as the primary site of fatty acid oxidation and lipoprotein synthesis, showed particularly strong lipid-associated methylation signals, consistent with its central role in whole-body lipid homeostasis [34,35].

### 4.3. Lipid Epigenetics and the Hallmarks of Aging

The correlation between methylation levels and maximum lifespan (Supplementary Figure S1) supports the “Epigenetic Maintenance” theory of aging [36,37]. Long-lived species appear to maintain these lipid-associated loci in a more stable state (either hyper- or hypomethylated) compared to short-lived species. This observation is consistent with recent findings that epigenetic clock CpGs are enriched near genes involved in development and that developmental processes may be mechanistically linked to aging [38,39].

Our finding that metabolic tissues such as liver and kidney exhibit more rapid age-related methylation drift (Figure 3) aligns with the “Disposable Soma” theory [40], where high-turnover metabolic organs accumulate epigenetic changes faster, potentially driving the systemic aging process. This accelerated epigenetic aging in metabolic tissues may reflect the cumulative burden of metabolic stress, including oxidative damage from mitochondrial respiration, exposure to dietary metabolites, and the constant demand for metabolic adaptation [41,42].

The relationship between lipid metabolism and aging is bidirectional. On one hand, aging is associated with characteristic changes in lipid profiles, including increased plasma triglycerides and altered fatty acid composition [43,44]. On the other hand, lipid metabolic dysfunction can accelerate biological aging, as evidenced by studies showing that epigenetic age acceleration is associated with dyslipidemia and cardiovascular disease risk [45,46]. Our results suggest that DNA methylation may serve as a molecular integrator of these bidirectional relationships, encoding long-term metabolic history and influencing future metabolic capacity.

Importantly, interventions known to extend lifespan, such as caloric restriction, have been shown to alter both lipid profiles and DNA methylation patterns at lipid-associated genes [47,48]. This suggests that the epigenetic regulation of lipid metabolism may be a modifiable target for interventions aimed at promoting healthy aging.

### 4.4. Implications for Metabolic Disease and Therapeutic Interventions

While sex hormones undoubtedly influence lipid levels, our data suggest that the fundamental epigenetic architecture is shared. The high male-female concordance (Figure 5) implies that these specific CpG sites regulate basal lipid homeostasis mechanisms required by both sexes, rather than sex-specific reproductive traits. This conservation across sexes strengthens the case that these epigenetic markers represent core, evolutionarily constrained mechanisms of metabolic regulation.

The genes related to lipid metabolism, particularly *CPT1A* and *ABCG1*, have been extensively studied in the context of metabolic disease [49,50]. Altered methylation at these loci has been associated with metabolic syndrome, type 2 diabetes, and cardiovascular disease in multiple human cohorts [51,52]. Our demonstration that these methylation patterns are evolutionarily conserved suggests that they may represent fundamental vulnerabilities or adaptive mechanisms that have been maintained across mammalian evolution.

From a therapeutic perspective, the reversibility of DNA methylation offers potential opportunities for intervention [53,54]. Dietary interventions, physical activity, and pharmacological agents have all been shown to influence DNA methylation patterns at metabolic genes [55,56]. Understanding the evolutionary conservation and tissue-specificity of these epigenetic responses may help in designing more targeted and effective interventions.

### 4.5. Study Limitations and Future Directions

Several limitations of our study should be acknowledged. First, our analysis focused on a limited number of lipid markers (HDL, TCH, TGY) and did not capture the full complexity of lipid metabolism, including specific lipid species such as ceramides, phospholipids, and oxidized lipids, which have also been implicated in aging [57,58]. Second, while we demonstrated correlations between methylation patterns and lifespan, our cross-sectional design cannot establish causality. Longitudinal studies tracking methylation changes within individuals and experimental manipulations in model organisms will be necessary to determine whether these epigenetic changes drive aging or are merely correlates.

Third, our tissue-level analysis does not capture cellular heterogeneity, which may be important given that different cell types within the same tissue can have distinct metabolic and epigenetic profiles [59]. Single-cell methylation technologies are beginning to address this limitation and will be valuable in future studies. Finally, while we demonstrated evolutionary conservation across mammals, we did not examine whether similar principles apply to non-mammalian vertebrates or invertebrates, which show different patterns of DNA methylation and metabolic regulation [60].

Future research should investigate the functional consequences of manipulating methylation at these conserved lipid-associated loci. CRISPR-based epigenetic editing tools now allow for targeted methylation changes without altering DNA sequence [61], providing a powerful approach to test causality. Additionally, integrating multi-omics data including transcriptomics, proteomics, metabolomics, and lipidomics will provide a more complete picture of how epigenetic variation at these loci influences phenotype across tissues and species.

## 5. Conclusions

By applying the Hybrid Pi-score to a pan-mammalian dataset, we demonstrated that lipid dysregulation is imprinted in the genome in a way that reflects evolutionary history, tissue function, and species longevity. Our results highlight the potential of these conserved epigenetic markers as universal metrics for biological aging and advocate for the use of blood as a surrogate for monitoring systemic metabolic health across species. The deep evolutionary conservation of lipid-associated epigenetic signatures suggests that these mechanisms are fundamental to mammalian biology and may represent promising targets for interventions aimed at promoting healthy aging and preventing age-related metabolic diseases.

## Figures and Tables

**Table 1:** Characteristics of top lipid-associated CpG sites. This table lists the top-ranked CpG sites selected based on their Hybrid Pi (HyPi) scores.

Probe ID	Sex	Chr	Location *	Count	Mean CR	Gene	Group	CpG Island
cg00271154	M	2	113033418	4	−0.014	*ZC3H6*	5′UTR	Island
cg00331096	M	20	22564947	4	0.032	*FOXA2*	5′UTR	Island
cg00585733	M	7	69064884	4	0.019	*AUTS2*	1stExon	Island
cg00838150	M	3	69788442	4	−0.01	*MITF*	TSS200	N_Shore
cg01125381	M	X	49644706	4	−0.011	*LOC158572*	TSS1500	S_Shore
cg02538199	M	9	37034277	6	−0.073	*PAX5*	1stExon	Island
cg04100724	M	12	92539208	4	−0.012	*BTG1*	1stExon	Island
cg04645342	M	1	72748124	4	−0.033	*NEGR1*	1stExon	N_Shore
cg06169648	M	16	85646832	4	−0.012	*KIAA0182*	TSS200	Island
cg08005992	M	11	31832959	4	−0.026	*PAX6*	TSS200	Island
cg09596336	M	2	145273297	4	0.028	*ZEB2*	Body	N_Shore
cg10414058	M	5	141017903	6	−0.078	*RELL2*	1stExon	S_Shore
cg14751398	M	6	20402153	4	0.025	*E2F3*	1stExon	Island
cg15735984	M	17	59477203	4	−0.018	*TBX2*	TSS200	Island
cg18634499	M	3	32022385	4	−0.179	*OSBPL10*	Body	Island
cg21097283	F	5	76934461	4	−0.174	*OTP*	5′UTR	N_Shore
cg26991812	F	17	27621123	4	−0.018	*NUFIP2*	5′UTR	N_Shore

* Genome assembly version: hg19.

## Data Availability

The R source code used for the analysis will be made available upon contacting the corresponding author. The KoGES data were obtained from the National Biobank of Korea, the Korea Disease Control and Prevention Agency, Republic of Korea (KBN-2024-045) and are available upon appropriate approval from the National Biobank. The mammalian methylation consortium data (GSE223748) are publicly available in the Gene Expression Omnibus database (https://www.ncbi.nlm.nih.gov/geo/query/acc.cgi?acc=GSE223748, accessed on 06 March 2026).

## Supplementary Materials

The following supporting information can be downloaded at: https://www.mdpi.com/article/10.3390/biomedicines14030597/s1, Figure S1: Association between species lifespan and methylation levels; Figure S2: Phylogenetic tree based on lipid-associated CpGs.

## Abbreviations

The following abbreviations are used in this manuscript:

CpG	Cytosine-phosphate-Guanine
HDL	High-Density Lipoprotein Cholesterol
HyPi	Hybrid Pi Score
KoGES	Korean Genome and Epidemiology Study
TCH	Total Cholesterol
TGY	Triglycerides
t-SNE	t-Distributed Stochastic Neighbor Embedding
