# Supplementary material for: Evolutionary Conservation of Lipid-Associated Epigenetic Signatures and Their Distinct Roles in Tissue Identity and Mammalian Aging
Source: Biomedicines. 2026 Mar 7;14(3):597. doi: 10.3390/biomedicines14030597 (PMC13024375; doi:10.3390/biomedicines14030597)
Supplement: Supplementary file 1 [file biomedicines-14-00597-s001.zip › biomedicines-4124076-supplementary.pdf]

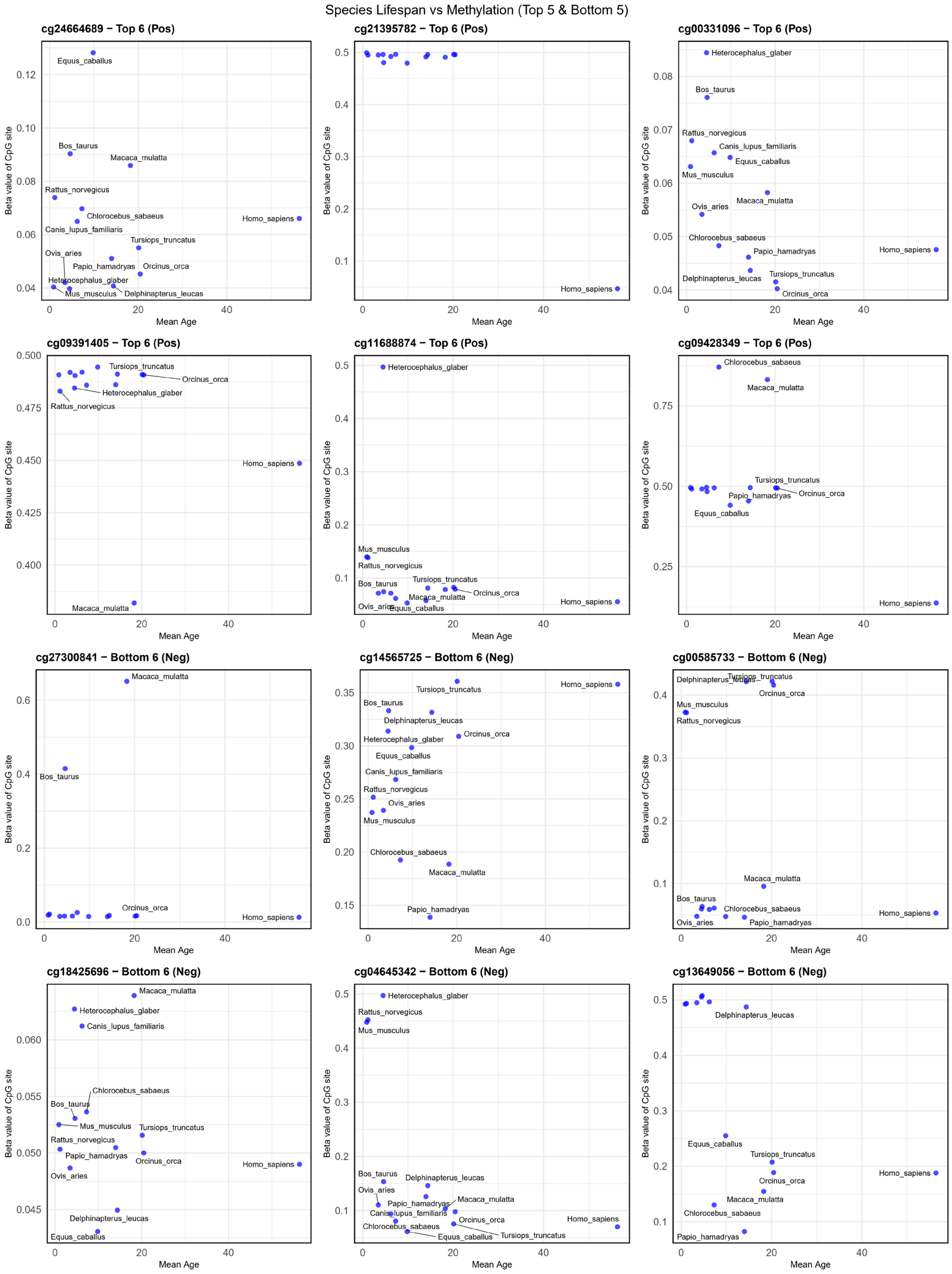

https://doi.org/10.3390/biomedicines14030597

Supplementary Figure S1. Association between species lifespan and methylation levels.

The correlation plots illustrate the relationship between the mean age (x-axis) and the mean beta values (y-axis) of the top and bottom six CpG sites, identified by their HyPi scores. A total of 14 mammalian species were analyzed, including primates (*H. sapiens*, *M. mulatta*, *P. hamadryas*, *C. sabaeus*), cetaceans (*O. orca*, *T. truncatus*, *D. leucas*), rodents (*M. musculus*, *R. norvegicus*, *H. glaber*), and other mammals (*B. taurus*, *O. aries*, *C. lupus familiaris*, *E. caballus*). Each point represents the mean value for a specific species, with labels provided for identification. Humans and long-lived cetaceans consistently display distinct methylation profiles compared to short-lived rodents. Notably, the naked mole-rat frequently appears as an outlier within the rodent group, exhibiting methylation patterns more similar to longer-lived species in several CpG sites, such as cg00331096 and cg11688874.

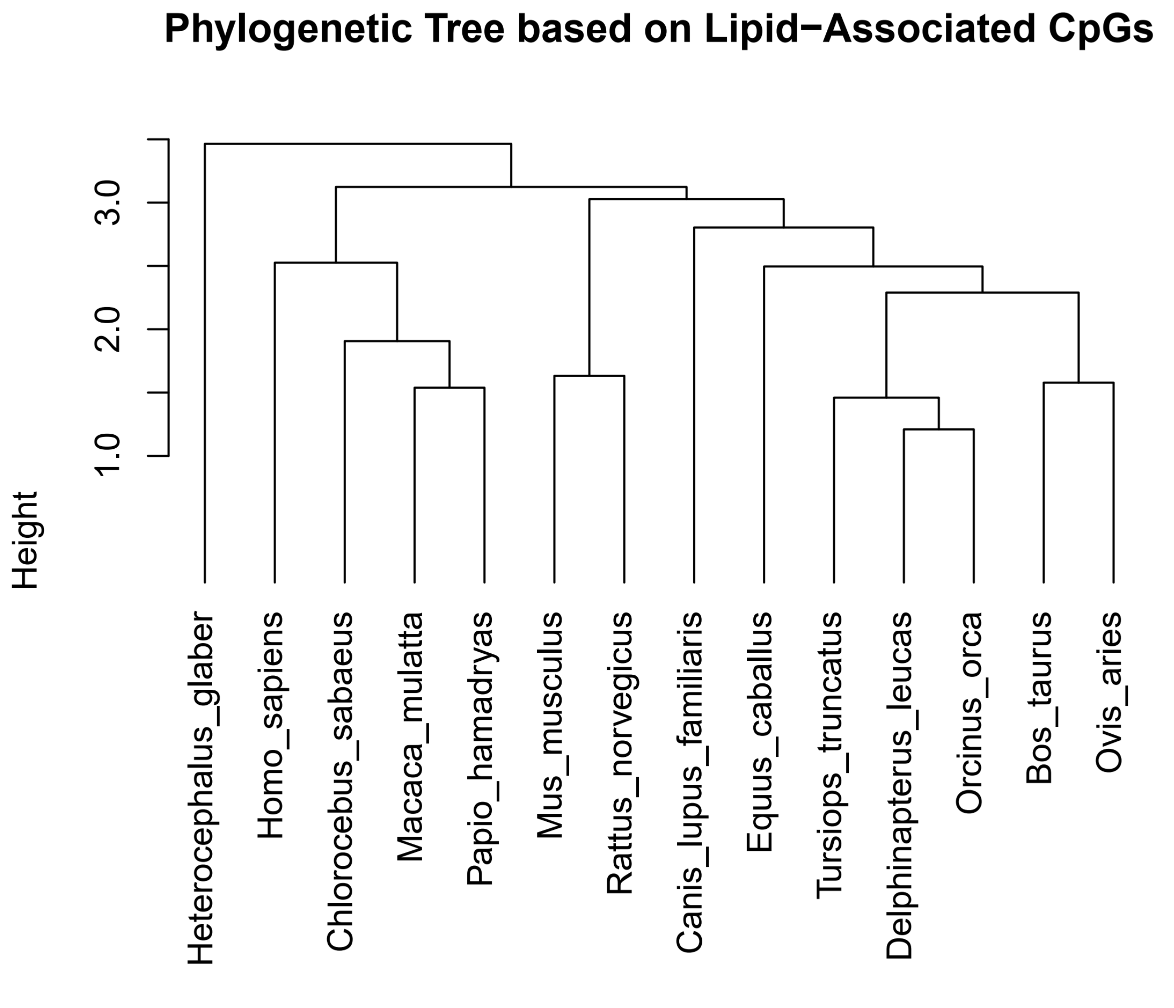

Supplementary Figure S2. Hierarchical clustering of 14 mammalian species based on lipid-associated CpG methylation profiles.

The dendrogram was constructed using Euclidean distances and the average linkage method based on the beta values of 239 lipid-associated CpG sites. Primates: Human (Homo sapiens), Rhesus macaque (Macaca mulatta), Hamadryas baboon (Papio hamadryas), and Green monkey (Chlorocebus sabaeus). Cetartiodactyla: Cattle (Bos taurus), Sheep (Ovis aries), Killer whale (Orcinus orca), Bottlenose dolphin (Tursiops truncatus), and Beluga whale (Delphinapterus leucas). Rodentia: House mouse (Mus musculus), Brown rat (Rattus norvegicus), and Naked mole-rat (Heterocephalus glaber). Others: Dog (Canis lupus familiaris) and Horse (Equus caballus). The clustering illustrates that humans share more similar lipid-related epigenetic signatures with other primates and cetaceans than with rodents, while the naked mole-rat shows a distinct separation from other rodent species.
